# Alterations in the inferior fronto-occipital fasciculus – a specific neural correlate of gender incongruence?

**DOI:** 10.1017/S0033291721005547

**Published:** 2023-06

**Authors:** Jason van Heesewijk, Martijn D. Steenwijk, Baudewijntje P. C. Kreukels, Dick J. Veltman, Julie Bakker, Sarah M. Burke

**Affiliations:** Center of Expertise on Gender Dysphoria, Amsterdam University Medical Centers, location VUmc, De Boelelaan 1131, Amsterdam, Noord-Holland, Netherlands

**Keywords:** Fractional anisotropy, gender incongruence, gender, gonadotropin-releasing hormone analogs, inferior fronto-occipital fasciculus, puberty, sex

## Abstract

**Background:**

Increasing numbers of adolescents seek help for gender-identity questions. Consequently, requests for medical treatments, such as puberty suppression, are growing. However, studies investigating the neurobiological substrate of gender incongruence (when birth-assigned sex and gender identity do not align) are scarce, and knowledge about the effects of puberty suppression on the developing brain of transgender youth is limited.

**Methods:**

Here we cross-sectionally investigated sex and gender differences in regional fractional anisotropy (FA) as measured by diffusion MR imaging, and the impact of puberty on alterations in the white-matter organization of 35 treatment-naive prepubertal children and 41 adolescents with gender incongruence, receiving puberty suppression. The transgender groups were compared with 79 age-matched, treatment-naive cisgender (when sex and gender align) peers.

**Results:**

We found that transgender adolescents had lower FA in the bilateral inferior fronto-occipital fasciculus (IFOF), forceps major and corpus callosum than cisgender peers. In addition, average FA values of the right IFOF correlated negatively with adolescents' cumulative dosage of puberty suppressants received. Of note, prepubertal children also showed significant FA group differences in, again, the right IFOF and left cortico-spinal tract, but with the reverse pattern (transgender > cisgender) than was seen in adolescents.

**Conclusions:**

Importantly, our results of lower FA (indexing less longitudinal organization, fiber coherence, and myelination) in the IFOF of gender-incongruent adolescents replicate prior findings in transgender adults, suggesting a salient neural correlate of gender incongruence. Findings highlight the complexity with which (pubertal) sex hormones impact white-matter development and add important insight into the neurobiological substrate associated with gender incongruence.

## Introduction

Gender identity (e.g. male, female, non-binary, genderqueer) is a uniquely human trait that develops during childhood (Halim & Ruble, 2010). Biological and sociocultural factors (family, peers, society) are thought to shape individual differences in the subjective experience of a child's gender (Fausto-Sterling, 2019). Gender identity is refined further during adolescence, a period of significant (physical, cognitive, and social-emotional) developmental changes. Within the context of the dramatic rise of pubertal sex hormone levels impacting an adolescent's body in a sex-specific way, and in relation to the emerging, yet not clearly formed sexual identity, gender identity is further differentiated (Ruble, Martin, & Berenbaum, 2006). For most individuals, birth-assigned sex and gender identity are overall congruent (i.e. cisgender). However, for individuals identifying as transgender, sex and gender do not align. Transgender individuals may meet the diagnostic criteria for gender incongruence (World Health Organization, 2018), which is defined as a marked feeling of incongruence between one's sex assigned at birth and one's experienced gender.

In recent years, exponential increases in referrals of adolescents to specialized gender identity services have been reported worldwide (Pang et al., 2020; Zucker, 2019). The ongoing increase in requests for medical treatments (such as puberty suppression and gender-affirming hormone treatment) of transgender youth asks for further examination of the development of gender identity and knowledge about the effects of puberty suppression on the developing brain.

A prominent hypothesis on the etiology of gender incongruence proposes that divergent early sexual differentiation mediates sex-atypical organization of the brain, and thereby the development of a gender identity not aligning with one's sex (Dörner, 1988; Swaab & Hofman, 1995; Zhou, Hofman, Gooren, & Swaab, 1995). Partial support for the sexual differentiation hypothesis, which was recently refined (Guillamon, Junque, & Gómez-Gil, 2016; Uribe et al., 2020), has since been provided by several magnetic resonance imaging (MRI) studies (Manzouri & Savic, 2019; also see reviews by Kreukels & Guillamon, 2016; Nguyen et al., 2019). One of these MRI measures, used to characterize white-matter microstructure by means of Diffusion Tensor Imaging (DTI), is fractional anisotropy (FA). Relatively higher overall, as well as region-specific FA values have been found in cisgender men compared to cisgender women (Bava et al., 2011; Cox et al., 2016; Den Braber et al., 2013; Hsu et al., 2008; Inano, Takao, Hayashi, Abe, & Ohtomo, 2011; Menzler et al., 2011; van Hemmen et al., 2016), indexing relatively more longitudinal organization, fiber coherence, and myelination in males, [but see Kochunov et al. (2015), who found higher FA values in females than males].

**Fig. 1.**
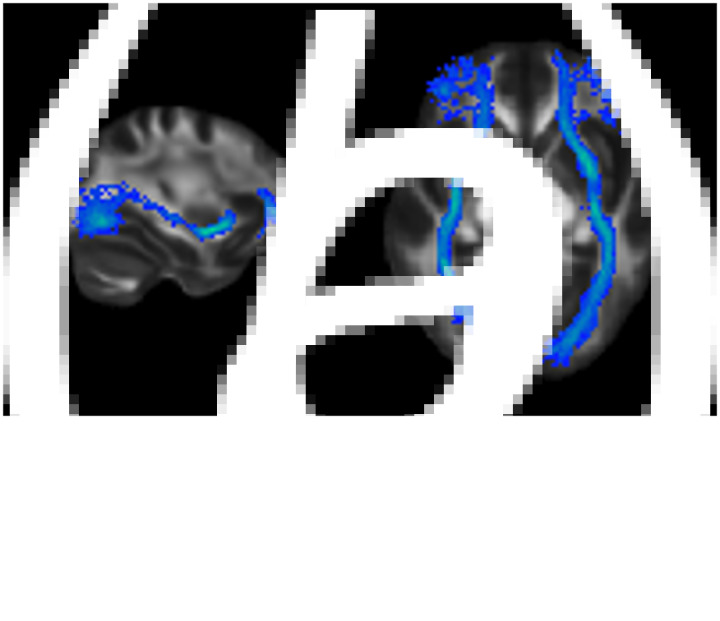
Anatomical representation of the Inferior Frontal Occipital Fasciculus in (*a*) sagittal and (*b*) transverse radiological display orientation. The bilateral IFOF was selected from the JHU white-matter tractography atlas (Mori, Wakana, van Zijl, & Nagae-Poetscher, 2005) and overlayed on the FMRIB58_FA_1 mm standard space image. The figure was created using FSL image viewer FSLeyes.

Comparing transgender individuals with cisgender controls, higher, thus masculinized FA values in a group of 18 transgender men (female sex assigned at birth, identifying as male) were found, whereas 18 transgender women (male sex assigned at birth, identifying as female) had intermediate FA values not showing significant differences from either of the cisgender groups (Rametti et al., 2011a; 2011b). Similarly, a study by Kranz et al. (2014) reported that transgender women and transgender men had parameters in between those of the two cisgender-control groups, thus diverging from their birth-assigned sex. More recently, sex by gender identity interaction in FA values was found specifically in the right inferior fronto-occipital fasciculus (IFOF, see Fig. 1 for an anatomical representation of the bilateral IFOF; Burke, Manzouri, & Savic, 2017). This tract connects occipital with frontal brain regions, carrying visual perceptual information to higher-order cognitive brain regions of the prefrontal cortex (Catani, 2006, 2007). The interaction effect revealed lower, thus sex-atypical FA values, in 27 transgender women compared with 29 homosexual and 40 heterosexual cisgender men (Burke et al., 2017). Interestingly, the effect was, thus, irrespective of sexual orientation. Therefore, the alterations in white-matter microstructure were specifically related to group differences in gender identity. However, in contrast to prior studies (Kranz et al., 2014; Rametti et al., 2011a, 2011b), no group differences were found in the birth-assigned females. Overall, the trans- *v.* cisgender group differences in earlier studies were found prior to any hormonal treatment, suggesting *a priori* sex-atypical differentiation of brain structures in adult individuals with gender incongruence.

In addition, pubertal development (Bava et al., 2011; Chahal et al., 2018; Genc et al., 2017; Schmithorst, Holland, & Dardzinski, 2008), as well as sex hormones (Herting, Maxwell, Irvine, & Nagel, 2012; Ho et al., 2020; Pangelinan et al., 2016; Peper et al., 2013; Peper, de Reus, van den Heuvel, & Schutter, 2015) have been differentially associated with white-matter diffusion characteristics in male and female adolescents. A 2-year longitudinal study in adolescents (two visits, age at first visit 10–18 years) showed FA *in*creases in cisgender boys and *de*creases in cisgender girls that were predicted by adrenal- and gonadal hormone-based changes (Herting et al., 2017). This suggests that puberty, independently of chronological age, influences brain development in a sex-specific way.

Lastly, a prospective case study of an 11-year-old transgender girl who received puberty suppression treatment, showed that the typical, testosterone-related white-matter maturation (i.e. increase in FA with older age) was not observed (Schneider et al., 2017). In addition, performance intelligence quotient and memory deteriorated over a period of 28 months. These single-case findings warrant further study of the effects of the puberty suppression treatment in transgender adolescents on white-matter development and cognitive functioning in larger samples.

In the current study, we investigated the hypothesized sex-atypical (Burke et al., 2017; Kranz et al., 2014; Rametti et al., 2011a, 2011b), and transgender-specific (Burke et al., 2017) differences in white-matter microstructure in relation to pubertal status, using a targeted region-of-interest approach. We included a sample of pre-pubertal children and adolescents who were referred to a gender-identity clinic, and applied identical analysis settings as in Burke et al. (2017) to ensure the highest possible comparability and to replicate previous findings in a younger, independent sample.

## Methods

### Participants

In a total of 82 children and 82 adolescents DTI data, among other (f)MRI paradigms (see Burke, Cohen-Kettenis, Veltman, Klink, & Bakker, 2014; Nota et al., 2017) were acquired. Due to insufficient data quality (because of e.g. artefacts, reconstruction errors; *N* = 4), insufficient data quality due to head motion (determined by visual inspection; *N* = 4), and lack of time to finish the MRI session^†^^1^ (*N* = 1), data of nine children had to be excluded, leaving datasets of 73 children and 82 adolescents for further analysis (see below for acquisition parameters).

Twenty prepubertal transgender girls (*M_age_* = 10.4, s.d. = 0.8), 15 prepubertal transgender boys (*M_age_* = 9.6, s.d. = 1.2), 20 adolescent transgender girls (*M_age_* = 15.4, s.d. = 1.1), and 21 adolescent transgender boys (*M_age_* = 16.1, s.d. = 0.8) were all diagnosed with gender incongruence-related diagnosis (at the time of inclusion the DSM-IV-TR criteria applied) and recruited via the Center of Expertise on Gender Dysphoria at the Amsterdam University Medical Centers, location VUmc in Amsterdam, the Netherlands. The prepubertal groups were treatment-naive at the time of the study. All adolescent participants with gender incongruence had been treated with monthly 3.75 mg Triptorelin (Decapeptyl-CR®, Ferring, Hoofddorp, the Netherlands) injections for, on average, 22.7 months (range 2–48 months) resulting in complete suppression of gonadal hormone production (Kreukels & Cohen-Kettenis, 2011).

The control groups, 18 prepubertal cisgender boys (*M_age_* = 9.5, s.d. = 1.0), 20 prepubertal cisgender girls (*M_age_* = 9.8, s.d. = 0.9), 20 adolescent cisgender boys (*M_age_* = 15.9, s.d. = 0.6) and 21 adolescent cisgender girls (*M_age_* = 16.3, s.d. = 1.0), were recruited via several secondary schools in the Netherlands, and by inviting friends of the participants with gender incongruence.

Details of the assessments of puberty stage, hormone analyses, MR image acquisition and preprocessing steps are described in the Supplementary Materials.

### Statistical analyses

#### Sample characteristics

Using the Statistical Package for the Social Sciences, version 25.0 (SPSS Inc., Chicago, IL, USA), we compared age between groups by means of one-way ANOVAs and Tanner stage between groups by means of chi-square tests. FA-residual distributions were tested for normality by means of Kolmogorov–Smirnov tests and residual histograms and Q-Q plots. All results were considered significant at *p* < 0.05 and/ or *η^2^* > 0.13 (medium effect size; Field, 2013) unless otherwise stated.

#### FA in relation to sex, gender, and pubertal status

To assess main and interaction effects of and between Sex, Gender and/or Pubertal status, we conducted a factorial multivariate analysis of variance (factorial MANOVA) with average FA values of all (*N* = 13) tracts as dependent variables, and Sex, Gender and Pubertal status (prepubertal; adolescent) as independent variables. Similar effects per tract were analyzed using separate univariate ANOVAs and *Post-hoc* one-way ANOVAs with planned contrasts were conducted to compare individual tract average FA values of transgender boys and girls with those of their birth-assigned-sex cisgender control group.

#### Covarying individual differences in pubertal stage

Pubertal stages (according to Marshall & Tanner, 1969, 1970) were added to the model as covariates in secondary analyses using Sex by Gender factorial MANOVA among the prepubertal and adolescent sub-sample separately. Univariate ANOVAs and *Post-hoc* independent factorial ANOVAs were again used to differentiate between tracts and sex/gender groups, respectively.

#### FA in relation to puberty suppression

Lastly, we conducted exploratory correlation analyses between average FA values (of those tracts that showed effects in the main analyses) and the cumulative doses of GnRHa received by the adolescent transgender girls and boys. Results were Bonferroni corrected for multiple testing (adjusted *α* = 0.05/4 tracts tested = 0.0125).

## Results

### Sample characteristics

Demographic information, including age, hormone levels, and Tanner stages for all groups is provided in Table 1. Among the children as well as the adolescents, significant age differences were found between groups, *F* (3, 68) = 3.0, *p* = 0.038, and *F* (3, 78) = 4.2, *p* = 0.008, respectively. However, *post-hoc* Bonferroni-corrected comparisons did not reveal significant age differences between groups of the same birth-assigned sex. By design, the groups differed in terms of Pubertal status. Almost all children were prepubertal and thus had Tanner stages of 1 for pubic hair and breast/genital development (see Table 1). Six children had Tanner stage 2 for pubic hair (three cisgender girls) *or* breast/genitals (one cisgender girl, one transgender girl, and one transgender boy), but were nevertheless included in the prepubertal group. All adolescents were pubertal [Tanner stages ranged from 2 to 6, except one transgender girl who had received GnRHa early on and had no pubic hair (Tanner P = 1)] and Tanner stages differed significantly between the groups for pubic hair (χ^2^ (15) = 36.9, *p* = 0.001) and breasts/genitals (χ^2^ (9) = 24.4, *p* = 0.004); transgender girls had significantly lower Tanner stages than the other adolescent groups.

**Table 1.** Subject characteristics per group

Children	Cisgender boys	Transgender girls	Transgender boys	Cisgender girls	*F*/χ^2^	*p*
*N*	18	20	15	20		
Age (years)	9.6 (1.0)	10.4 (0.8)	9.6 (1.2)	9.7 (0.9)	3.0	0.038*
Age range	7.8–11.4	8.3–12.0	7.8–11.3	8.1–11.8		
LH (IU/mmol)	0.01 (0.01)	0.04 (0.11)	0.00 (0.02)	0.01 (0.03)		
FSH (IU/mmol)	0.16 (0.08)	0.37 (0.25)	0.32 (0.23)	0.44 (0.34)		
DHEA (*μ*mol/mmol)	0.00 (0.02)	0.01 (0.02)	0.01 (0.02)	0.00 (0.01)		
Tanner P	1 (1–1)	1 (1–1)	1 (1-1)	1 (1–2)^$^	8.1	0.**043**
Tanner M or G	1 (1–1)	1 (1–2)^$^	1 (1-2)^$^	1 (1–2)^$^	1.1	0.775
Adolescents	Cisgender boys	Transgender girls	Transgender boys	Cisgender girls		
*N*	20	20	21	21		
Age (years)	15.9 (0.6)	15.6 (1.1)	15.9 (0.8)	16.3 (1.0)	4.2	0.008*
Age range	14.9–17.3	12.8–18.2	15.1–18.0	14.5–19.3		
Testosterone (pmol/l)	307.0 (106.5)	–	–	40.5 (60.4)		
Estradiol (pmol/mmol)	245.3 (183.1)	–	–	529.6 (643.3)		
Cumulative GnRHa dose (mg)	–	88.1 (46.2)	78.8 (60.8)	–		
Tanner P	5 (3–6)	3 (1–5)	5 (3–6)	4 (3–5)	36.9	0.**001**
Tanner M or G	4 (2–5)	3 (2–5)	5 (2–5)	4 (3–5)	24.4	0.**004**

Continuous data are presented as median (s.d.), Tanner stages as median (range). Luteinizing hormone (LH), follicle-stimulating hormone (FSH), dehydroepiandrosterone (DHEA), and Estradiol are corrected for creatinine. Tanner P = pubic hair stage, Tanner M or G = breast or genital stage, *Post-hoc Bonferroni corrected comparisons revealed no age differences between the same birth-assigned sex groups (i.e. cisgender boys v. transgender girls, and cisgender girls v. transgender boys), only adolescent transgender girls were significantly younger than adolescent cisgender girls. ^$^Six children had Tanner stage 2 for pubic hair (three cisgender girls) *or* breast/genitals (one cisgender girl, transgender girl, and transgender boy). Bold typeface = *p* <0.05.

### FA in relation to sex, gender, and pubertal status

FA values per group and tract are provided in the supplementary materials – Table S2. Assumptions for normality of FA-residual distributions were met. The Sex (birth-assigned female; male) by Gender (female; male) by Pubertal status (prepubertal; adolescent) factorial MANOVA revealed, using Wilks' Lambda, an overall (across all tracts) significant main effect of Pubertal status, ∧ = 0.10, *F* (13, 135) = 98.2, *p* < 0.001. An interaction effect of Sex and Gender (∧ = 0.82, *F* (13, 135) = 2.1, *p* = 0.012) showed that, across all tracts examined and in both age groups, cisgender and transgender individuals differed in the white-matter organization. An additional three-way interaction effect of Pubertal status, Sex and Gender (∧ = 0.83, *F* (13, 135) = 2.1, *p* = 0.018) indicated that the pattern of cis- *v.* transgender group differences was different for the prepubertal *v.* adolescent groups. No overall main effects of Sex or Gender, and no interaction effect between Pubertal status and Sex, or Pubertal status and Gender were found.

**Fig. 2.**
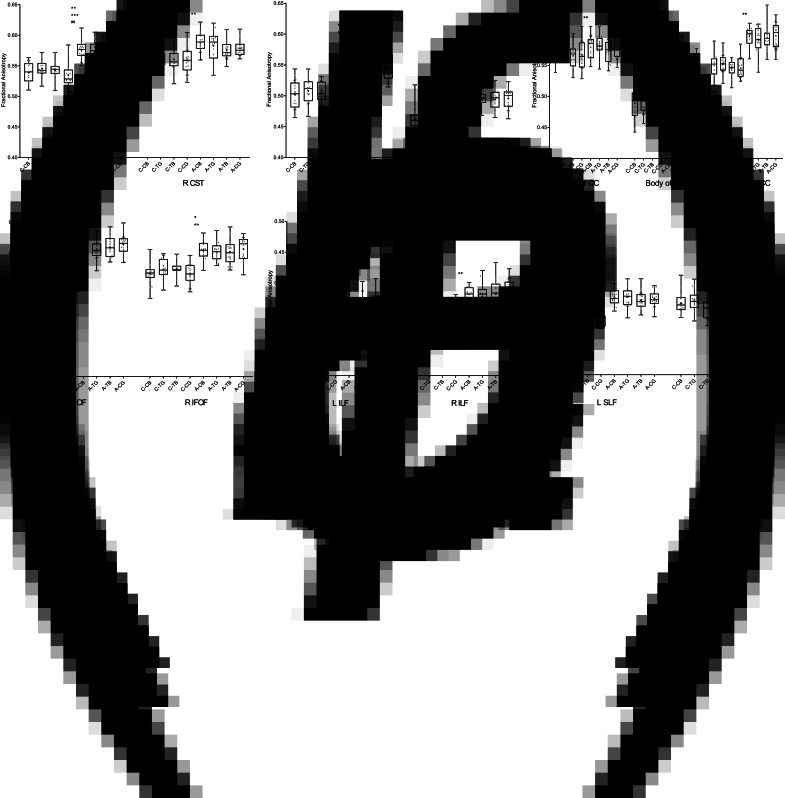
Boxplots showing fractional anisotropy (FA) values per tract (*a*–*f*). Group differences were analyzed with: (1) Sex (birth-assigned female/male) by Gender identity (female/male) by Pubertal status (prepubertal/adolescent) factorial MANOVA including all cisgender and transgender groups *without* covariates. Here * denotes significant three-way interaction effects of Sex, Gender and Pubertal status (LIFOF, *F* (1, 147) = 7.4, *p* = 0.007, *η*^2^ = 0.05; RIFOF, *F* (1, 147) = 4.5, *p* = 0.036, *η*^2^ = 0.03; forceps major, *F* (1, 147) = 4.6, *p* = 0.033, *η*^2^ = 0.03), ** main effects of Pubertal status (LCST, *F* (1, 147) = 106.3, *η*^2^ = 0.42; RCST, *F* (1, 147) = 51.9, *η*^2^ = 0.26; Forceps major, *F* (1, 147) = 150.8, *η*^2^ = 0.51; Forceps minor, *F* (1, 147) = 183.9, *η*^2^ = 0.56; LIFOF, *F* (1, 147) = 371.4, *η*^2^ = 0.72; RIFOF, *F* (1, 147) = 150.3, *η*^2^ = 0.51; LILF, *F* (1, 147) = 412.7, *η*^2^ = 0.74; RILF, *F* (1, 147) = 136.9, *η*^2^ = 0.48; LSLF, *F* (1, 147) = 280.7, *η*^2^ = 0.66; RSLF, *F* (1, 147) = 149.9, *η*^2^ = 0.51; Genu of CC, *F* (1, 147) = 13.6, *η*^2^ = 0.09; Body of CC, *F* (1, 147) = 68.4, *η*^2^ = 0.32; Splenium of CC, *F* (1, 147) = 271.0, *η*^2^ = 0.65. All *p* < 0.001), and *** main effects of Sex (LCST, *F* (1, 147) = 6.1, *p* = 0.015, *η*^2^ = 0.04; RSLF, *F* (1, 147) = 4.2, *p* = 0.042, *η*^2^ = 0.03). (2) *Post-hoc* one-way ANOVA comparing groups of same birth-assigned sex for children and adolescents separately with denoting a significant difference between indicated groups (LIFOF, *t* (147) = 2.7, *p* = 0.008). (3) Sex (birth-assigned female/male) by Gender (female/male) factorial MANOVA including adolescents only with Tanner stages (pubic hair growth, breast and genital development) as covariates. Here **# denotes an interaction effect of Sex and Gender** (Body of CC, *F* (1, 73) = 5.2, *p* = 0.026, *η*^2^ = 0.07), and **## a main effect of Sex** (LCST, *F* (1, 73) = 7.8, *p* = 0.032, *η*^2^ = 0.06). All results were considered significant at *p* < 0.05 and/or *η*^2^ > 0.13, + denotes mean FA per group. C-, child; A-, adolescent; CB, cisgender boy; TG, transgender girl; TB, transgender boy; CG, cisgender girl; L, left; R, right; CST, cortico-spinal tract; CC, corpus callosum; IFOF, inferior fronto-occipital fasciculus; ILF, inferior longitudinal fasciculus; SLF, superior longitudinal fasciculus. For illustrative purposes, *Y*-axis range is different for *a*–*c* and *d*–*f*.

Separate univariate ANOVAs on the tracts showed effects of Pubertal status in all tracts, with higher FA values in adolescents than in children. In contrast to the overall effects, no interaction effect between Sex and Gender was revealed in any of the tracts separately, and a main effect of Sex was shown for two major long white-matter tracts; right superior longitudinal fasciculus (RSLF), *F* (1, 147) = 4.2, *p* = 0.042, *η*^2^ = 0.03, and left cortico-spinal tract (LCST), *F* (1, 147) = 6.1, *p* = 0.015, *η*^2^ = 0.04, with higher FA values in birth-assigned boys compared to birth-assigned girls. No effect of Gender was revealed, nor an interaction effect of Sex and Gender, Pubertal status and Sex, or Pubertal status and Gender (see Fig. 2*a*–*f*).

Interestingly, a three-way interaction of Pubertal status, Sex and Gender was significant for the left IFOF (LIFOF), *F* (1, 147) = 7.4, *p* = 0.007, *η*^2^ = 0.05, right IFOF (RIFOF), *F* (1, 147) = 4.5, *p* = 0.036, *η2* = 0.03, and forceps major, *F* (1, 147) = 4.6, *p* = 0.033, *η*^2^ = 0.03. As displayed in Fig. 2*b* and 2*d*, adolescent cisgender individuals had higher FA values than their transgender peers, whereas the opposite pattern -relatively higher FA in trans- *v.* cisgender was observed in the prepubertal children. *Post-hoc* one-way ANOVAs with planned contrasts showed significantly higher average FA values in LIFOF in the adolescent cisgender boys than transgender girls, *t* (147) = 2.7, *p* = 0.008 (Fig. 2*d*). This pattern of FA value group differences (adolescent cisgender boys > transgender girls) was not observed in RIFOF and forceps major. No differences were found between the prepubertal cisgender boys and transgender girls, *t* (147) = −1.2, *p* = 0.215 (Fig. 2*d*), thus the difference between the cis- and transgender birth-assigned males was significant only in adolescence. Also, neither the prepubertal, nor adolescent birth-assigned females showed any significant group differences (Fig. 2*b* + *d*).

### Covarying individual differences in pubertal stage

Tanner stages for pubic hair growth differed significantly among the **prepubertal children** (see Table 1). Therefore, we conducted an additional Sex by Gender factorial MANOVA among the prepubertal sub-sample, adding Tanner P stages as a covariate. We found an interaction effect across tracts, indicating that transgender children had overall *higher* FA values than the cisgender boys and girls, ∧ = 0.62, *F* (13, 55) = 2.6, *p* = 0.008, *η*^2^ = 0.38, when accounting for Pubertal status. The overall main effects of Sex and Gender were not significant, but had medium effect sizes; *η2* = 0.16 and *η*^2^ = 0.14, respectively. Separate univariate ANOVAs confirmed interactions between Sex and Gender in specifically the RIFOF, *F* (1, 67) = 5.7, *p* = 0.019, *η*^2^ = 0.08 and LCST, *F* (1, 67) = 4.3, *p* = 0.042, *η*^2^ = 0.06. A main effect of Sex was significant for the RSLF, *F* (1, 67) = 4.9, *p* = 0.030, *η*^2^ = 0.07, with birth-assigned males having higher FA values than birth-assigned females. There was no significant main effect of Gender for any of the tracts. *Post-hoc* independent factorial ANOVAs with planned contrasts showed no difference in average FA values for RIFOF or LCST between transgender girls and cisgender boys, or transgender boys and cisgender girls.

Similar as for the children, Tanner stages differed significantly among the **adolescent groups**. Therefore, we conducted an additional Sex by Gender factorial MANOVA with adolescents only and both Tanner stages (P and M/G) as covariates (these were missing for three transgender girls). This analysis revealed an overall main effect of Sex, ∧ = 0.64, *F* (13, 61) = 2.7, *p* = 0.005, *η*^2^ = 0.36, with higher FA values in birth-assigned males than females. No significance, but medium and large effect sizes were found for an overall (across all tracts) main effect of Gender, *η*^2^ = 0.16, and an overall interaction effect of Sex and Gender, *η*^2^ = 0.27, respectively. Separate univariate ANOVAs on the tracts revealed a significant main effect of Sex in left CST, *F* (1, 73) = 7.8, *p* = 0.032, *η*^2^ = 0.06. No main effect of Gender was found. However, now an interaction effect of Sex and Gender reached significance in the body of the corpus callosum, *F* (1, 73) = 5.2, *p* = 0.026, *η*^2^ = 0.07, indicating that, when accounting for pubertal maturation differences, adolescent cisgender boys and girls had higher FA values than adolescent transgender boys and girls in this tract. *Post-hoc* independent factorial ANOVAs with planned contrasts showed an FA value difference in the body of the corpus callosum of 0.017, *p* = 0.031, 95% CI 0.002–0.032 between cisgender girls and transgender boys with higher values in cisgender girls. No difference was found between cisgender boys and transgender girls.

### FA in relation to puberty suppression

**Fig. 3.**
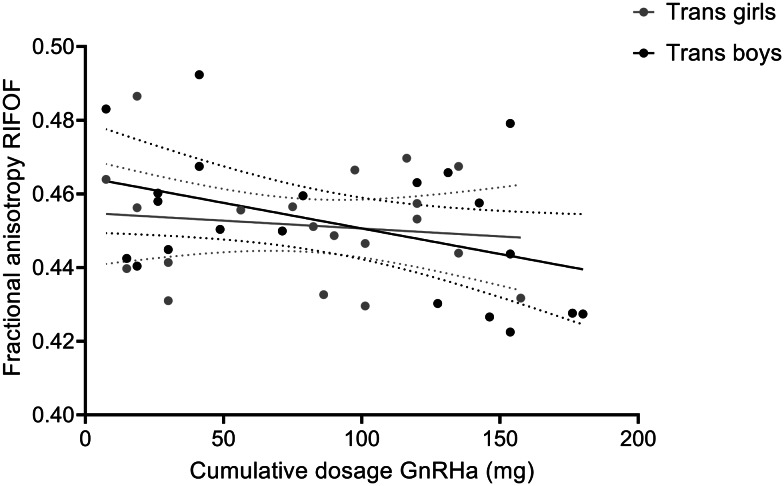
Correlation between of fractional anisotropy (FA) values of the RIFOF and cumulative doses of GnRHa received by the adolescent transgender boys and girls. Data are presented with 95% confidence bands. Correlation analysis including all transgender adolescents revealed a negative association between FA values and the cumulative doses of GnRHa (mg), *r* = −0.322, two-sided, uncorrected *p* = 0.040, *N* = 41. For illustrative purposes, data points for transgender boys and girls are shown in different gray shades.

The adolescent participants with gender incongruence (transgender boys and transgender girls) were the only two groups that had received GnRHa for suppression of endogenous puberty, while the cisgender control groups and the prepubertal children were all treatment-naive. In order to explore whether our findings of relatively lower FA in the adolescent transgender groups might be explained by the GnRHa treatment, we conducted correlation analyses between FA values and the cumulative doses of GnRHa received. These analyses revealed a negative association between FA values and the cumulative doses of GnRHa for the RIFOF; the more GnRHa received, the lower FA values were (*r* = −0.322, two-sided, uncorrected *p* = 0.040, *N* = 41; see Fig. 3). This effect, however, did not survive Bonferroni correction for multiple testing (adjusted *α* = 0.05/4 tracts tested = 0.0125). For the body of the corpus callosum, the LIFOF, and the forceps major no correlations between FA and the cumulative doses of GnRHa received were found.

## Discussion

The present study investigated potential sex-atypical (Burke et al., 2017; Kranz et al., 2014; Rametti et al., 2011a, 2011b) and transgender-specific (Burke et al., 2017) differences in white-matter microstructure in relation to pubertal status. By using the same analytical approach as Burke et al. (2017); comparing average FA values in white-matter tracts of interest across groups, the present study aimed to replicate and compare (as much as possible) the current findings in younger samples with those of the prior study in adults. Including both prepubertal and adolescent samples further enabled us to highlight the importance of puberty and adolescence in sex- and gender-specific white-matter microstructure.

**Fig. 4.**
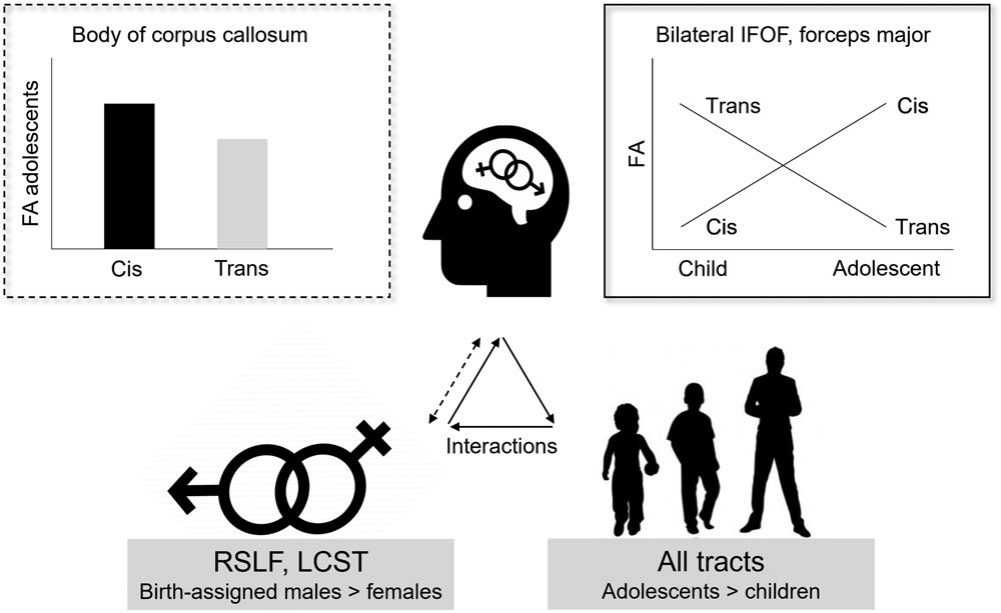
Infographic summarizing all main results (from upper left to lower right side): (1) Interaction effect of Sex and Gender (Body of corpus callosum, *F* (1, 73) = 5.2, *p* = 0.026, *η*^2^ = 0.07), (2) three-way interaction effects of Sex, Gender and Pubertal status (L IFOF, *F* (1, 147) = 7.4, *p* = 0.007, *η*^2^ = 0.05; R IFOF, *F* (1, 147) = 4.5, *p* = 0.036, *η*^2^ = 0.03; forceps major, *F* (1, 147) = 4.6, *p* = 0.033, *η*^2^ = 0.03), (3) main effects of Sex (R SLF, *F* (1, 147) = 4.2, *p* = 0.042, *η*^2^ = 0.03; L CST, *F* (1, 147) = 6.1, *p* = 0.015, *η*^2^ = 0.04), (4) main effects of Pubertal status (L CST, *F* (1, 147) = 106.3, *η*^2^ = 0.42; R CST, *F* (1, 147) = 51.9, *η*^2^ = 0.26; Forceps major, *F* (1, 147) = 150.8, *η*^2^ = 0.51; Forceps minor, *F* (1, 147) = 183.9, *η*^2^ = 0.56; L IFOF, *F* (1, 147) = 371.4, *η*^2^ = 0.72; R IFOF, *F* (1, 147) = 150.3, *η*^2^ = 0.51; L ILF, *F* (1, 147) = 412.7, *η*^2^ = 0.74; R ILF, *F* (1, 147) = 136.9, *η*^2^ = 0.48; L SLF, *F* (1, 147) = 280.7, *η*^2^ = 0.66; R SLF, *F* (1, 147) = 149.9, *η*^2^ = 0.51; Genu of CC, *F* (1, 147) = 13.6, *η*^2^ = 0.09; Body of CC, *F* (1, 147) = 68.4, *η*^2^ = 0.32; Splenium of CC, *F* (1, 147) = 271.0, *η*^2^ = 0.65. All *p* < 0.001). FA, fractional anisotropy; cis, cisgender; trans, transgender; L, left, R,  right; CST, cortico-spinal tract; IFOF, inferior fronto-occipital fasciculus; ILF, inferior longitudinal fasciculus; SLF, superior longitudinal fasciculus.

In line with several previous studies (Bava et al., 2011; Cox et al., 2016; Den Braber et al., 2013; Hsu et al., 2008; Inano et al., 2011; Menzler et al., 2011; van Hemmen et al., 2016), we found significant sex effects on FA group differences across age groups, with birth-assigned males showing higher FA values than birth-assigned females in the left CST and right SLF (see Fig. 4 for a summary of all main results). Though not significant for individual tracts, there was an overall significant interaction effect of Sex and Gender, suggesting general differences between trans- and cisgender groups and across both age groups. Interestingly, a significant three-way interaction effect of pubertal status, gender, and sex indicated that specific trans- *v.* cisgender group differences in FA were dependent on participants' sex *and* pubertal status; in the body of the corpus callosum both adolescent transgender groups had significantly reduced FA, and in the left IFOF adolescent transgender girls had significantly lower, thus *sex-atypical*, FA compared with the adolescent cisgender boys. Note however, that effect sizes were small.

Strikingly, these latter results are highly similar to those of Burke et al. (2017). In that study, six groups of adult participants were compared; cisgender homosexual and cisgender heterosexual men and women, as well as transgender men and women with diverse sexual orientations (total *N* = 206). It was found that both the transgender and the cisgender *homosexual* (reference for both is the sex assigned at birth) groups had sex-atypical average FA values in several white-matter tracts, indicating less pronounced sexual differentiation. Interestingly, when individual differences in sexual orientation were controlled for (by adding scores on the sexual orientation questionnaire as a covariate), significantly lower FA in the right IFOF was specifically found in the transgender women, compared with the cisgender -heterosexual *and* homosexual- men. Thus, a transgender girl/ woman *v.* cisgender boy/ man difference in FA was found to be confined to the IFOF, both in the current study and that by Burke et al. (2017).

### The interplay of pubertal status, gender incongruence, and alterations in FA

Interestingly, in the prepubertal sample, we observed higher FA in children with gender incongruence compared with their age-matched cisgender peers. This novel observation is difficult to interpret, due to the lack of comparison literature. Therefore, future longitudinal research investigating how this reversed pattern in childhood can be explained is warranted.

Even though our cross-sectional data limit any conclusions regarding brain *development,* this study suggests that the years between childhood and mid adolescence, hence early adolescence, represent a highly important period in which puberty-related factors influence white-matter development in both a sex- and gender identity-specific way. Our findings are complementary to previous longitudinal studies in cisgender adolescent samples, which found sex differences in FA to be associated with puberty and increasing gonadal hormone levels (Herting et al., 2012, 2017), particularly of testosterone (Ho et al., 2020).

According to clinical treatment protocols (Coleman et al., 2012; Hembree et al., 2017), puberty suppression treatment is recommended for adolescents who have reached or are beyond the early stages of puberty (Tanner II-III). Thus, our 16-year-old participants had been exposed to endogenous sex hormones for at least some time, before they had started puberty-suppression treatment. Adult participants in the study by Burke et al. (2017) were treatment-naive, thus they had not received GnRHa and also had been exposed to endogenous sex steroids for several years. Therefore, we speculate that the post-pubescent cis- *v.* transgender group differences may be due to activational effects, of in particular testosterone. Early pubertal changes in testosterone levels, already before the start of puberty suppression treatment, might thus differently affect white-matter development in trans- *v.* cisgender youth. Future longitudinal studies should address the question how hormonal changes during puberty affect the development of gender identity and whether (variations in) gender identity, rather than sex *per se*, might interact with these associations of pubertal and brain development. In particular, the role of testosterone should be examined since this was impossible in the current study, because such data were not available in the transgender groups.

### Puberty and puberty suppression in relation to FA

A marked result of the present study was the significant pubertal-status effect in all tracts. Of note, while all adolescents showed higher FA compared to the children, patterns of average FA differences between cisgender and transgender groups reversed across development. More specifically, the cisgender individuals seemed to show a steeper ‘increase’ in FA, resulting in the pattern of adolescent cisgender > transgender group differences.

Suppression of puberty with GnRHa has become the treatment of choice for gender incongruence in youth (Coleman et al., 2012; Hembree et al., 2017). Its aim is to halt the development of the secondary sex characteristics, and it has been shown to significantly improve adolescents' mental health and well-being (Costa et al., 2015; van der Miesen, Steensma, de Vries, Bos, & Popma, 2020). However, there have been concerns that long-term delay of puberty with GnRHa, and thus prevention of exposure to sex hormones during the early adolescent years could interfere with significant neuro-developmental changes, particularly within the prefrontal cortex, which underlie adolescence-specific changes in behavior (e.g. in behavioral control and social cognition; Chen et al., 2020; Griffin, Clyde, Byng, & Bewley, 2020). Both pubertal stage and timing of pubertal onset have been found to influence brain development (Herting & Sowell, 2017; Juraska & Willing, 2017). Therefore, the effects of puberty suppression on the brain and cognitive development of youth with gender incongruence should be examined. Indeed, a recent study in mice suggested sex-specific adverse effects of GnRHa on stress-processing, mood and cognition (in females) and locomotion and social behavior (in males; Anacker et al., 2020). Similarly, preliminary evidence from a longitudinal case study suggested adverse effects of GnRHa on the brain and cognition in humans (Schneider et al., 2017). However, in our cross-sectional dataset of transgender adolescents, we did not find significant associations between FA and cumulative doses of GnRHa received. In addition, a study by Staphorsius et al. (2015) showed no effect of GnRHa treatment on executive functioning, measured with the Tower of London task, in 20 transgender adolescents. Systematic, well-powered studies, testing the long-term effects of puberty suppression on the brain and behavior should further investigate these early findings and whether gender-affirming hormone treatment restores potential alterations. Importantly, without puberty suppression, socio-emotional consequences of increased feelings of gender incongruence could also elicit alterations.

### The IFOF – neural correlate of gender incongruence?

Relatively lower FA in specifically the IFOF has been linked to gender incongruence in both adolescents (this study) and adults (Burke et al., 2017). In addition, in a longitudinal study of adult transgender men, testosterone treatment was associated with an *increase* of FA in specifically the IFOF (Burke et al., 2018). How is this particular white-matter tract, in interaction with sex-hormone changes, associated with gender incongruence?

The IFOF (among other tracts) has been suggested to be involved in social-emotional stimuli processing, emotional face recognition, emotion regulation, and attention (Catani & Thiebaut de Schotten, 2008; Doricchi, Thiebaut de Schotten, Tomaiuolo, & Bartolomeo, 2008; Philippi, Mehta, Grabowski, Adolphs, & Rudrauf, 2009; Taddei, Tettamanti, Zanoni, Cappa, & Battaglia, 2012). A longitudinal study showed that poor performance during an emotional face perception task at 7–9 years of age, was predictive of reduced FA of this ‘ventral-limbic white-matter pathway’ at age 14–15 years (Taddei et al., 2012). Prior studies have provided preliminary evidence of adult transgender men being less accurate than cisgender females in a visual face processing task (Feusner et al., 2016), but could not directly link this behavioral finding to neurobiological group differences.

Several studies have suggested an association between reduced FA in the IFOF and psychiatric conditions, such as generalized anxiety disorder (Liao et al., 2014), obsessive-compulsive disorder (Garibotto et al., 2010), and body dysmorphic disorder (Buchanan et al., 2013). Furthermore, lower FA in the IFOF has been linked to non-clinical anxious personality traits (Lu, Yang, Chu, & Wu, 2018) and was recently proposed to reflect a more ‘general psychopathology marker’ (Riem et al., 2019). Important to note, gender incongruence is not pathological. However, experiencing gender incongruence may come with disadvantages leading to an increased risk of mental health problems. These include, among others, minority stress, societal inequities, and barriers to healthcare, e.g., due to long waiting lists for transgender-specific healthcare, lack of physicians' knowledge, or discrimination in healthcare encounters (Braun, Garcia-Grossman, Quinones-Rivera, & Deutsch, 2017; Koehler, Strauss, Briken, Szuecs, & Nieder, 2021; Lo & Horton, 2016). In line with this, gender diversity in the general population has been associated with adolescent psychopathology (Burke, 2020; Potter et al., 2020) and young individuals with gender incongruence show elevated rates of diverse mental health problems and psychiatric comorbidity, especially before the start of treatment (Aitken, VanderLaan, Wasserman, Stojanovski, & Zucker, 2016; de Vries, Doreleijers, Steensma, & Cohen-Kettenis, 2011; Griffin et al., 2020; Holt, Skagerberg, & Dunsford, 2016; Kaltiala-Heino, Sumia, Työläjärvi, & Lindberg, 2015).

Thus, brain structural alterations in the IFOF seem to be a common neurobiological denominator of (vulnerability toward) psychopathology and sex-gender incongruence. Future longitudinal studies should further investigate which neurobiological, psycho-developmental, and societal context-related factors are predictive of adolescent mental health problems, also gender incongruence.

### Strengths and limitations

A strength of the present study is the inclusion of the unique sample of prepubertal children and adolescents with gender incongruence and the hypothesis-driven analysis approach. However, because of the relatively small sample sizes and effect sizes in some analyses, generalizability of results may be limited. Also, our cross-sectional design prohibits any conclusions regarding causality. Therefore, cautious interpretation and longitudinal replication of these findings are warranted. In addition, when averaging FA values, we did not apply masking, therefore, it is not certain that only white matter was taken into account during this process. Lastly, all FA results in frontal areas might have been influenced by the lack of top up and down epi-distortion correction.

## Conclusion

Taken together, in the present study we replicated the finding of lower FA in the IFOF of post-pubescent transgender girls and women, which suggests this to be a salient neural correlate of gender incongruence. In addition, we demonstrate that these adolescence-specific trans- *v.* cisgender group differences in FA show the reverse pattern in childhood. This highlights the complexity with which sex hormones, during different phases of human development, interact with and impact psychological and brain development, including the gendered perception of self. Our findings add important insights into the neurobiological substrate associated with gender incongruence.

## Footnotes

† The notes appear after the main text.

## Note

1 Diffusion tensor imaging data acquisition was scheduled at the end of the scanning protocol and the MR session already exceeded the time allotted.

## Supplementary material

For supplementary material accompanying this paper visit https://doi.org/10.1017/S0033291721005547. click here to view supplementary material
