# Supplementary material for: Alterations in the inferior fronto-occipital fasciculus – a specific neural correlate of gender incongruence?
Source: Psychol Med. 2022 Mar 18;53(8):3461–70. doi: 10.1017/S0033291721005547 (PMC10277722; doi:10.1017/S0033291721005547)
Supplement: Supplementary file 1 [file S0033291721005547sup001.docx]

**Supplementary Materials**

**Methods**

**Assessment of puberty stage**

All (cisgender and gender-incongruent) children underwent a short physical assessment by a pediatric endocrinologist to ensure their prepubertal status. In adolescent participants with gender incongruence, as part of their regular medical check-ups at the clinic, pubertal stages (pubic hair growth; Tanner P, breast and genital development; Tanner M/G, respectively) were determined by a pediatric endocrinologist as well. In the adolescent cisgender groups, pubertal staging was assessed by means of self-report on a five-point (1 = prepubertal, 5 = postpubertal) Tanner Maturation Scale participants were shown drawings of the different developmental stages and were asked to indicate which drawing fitted their stage best (Marshall & Tanner, 1969, 1970). These self-reports have been shown to correlate highly with physician assessments (Duke, Litt, & Gross, 1980; Morris & Udry, 1980).

**Hormone assessments**

Participants were asked to collect saliva and urine samples at home, directly after waking up at the day of the MR examination. Samples were brought to the clinic and stored at -80˚C until analysis. Dehydroepiandrosterone (DHEAs), follicle-stimulating hormone (FSH), and luteinizing hormone (LH) levels were measured in all children. In the cisgender adolescent groups, we determined estradiol and testosterone levels. Because in the transgender adolescents sex hormones were suppressed by gonadotropin releasing hormone analogues (GnRHa), and thus under detection threshold (Soleman et al., 2013), no assessments of their sex-hormone levels were done. Therefore, saliva samples were not used for this group, and neither for the prepubertal participants.

LH and FSH levels were measured in urine by immunometric assays (Architect, Abbott Laboratories Diagnostics Division, Abbott Park, Illinois, USA) as described before (Kuijper et al., 2006). For FSH lower limit of quantitation is 0.11 IU/L, intra-assay coefficient of variation is 3% at 5.5 IU/L, 25 IU/L and 75 IU/L and inter-assay coefficients of variation are 6% at 5 IU/L and 5% at 18 IU/L. For LH lower limit of quantitation is 0.1 IU/L, intra- assay coefficient of variation is 3% at 5 IU/L, 40 IU/L and 75 IU/L and inter-assay coefficients of variation are 7% at 4 IU/L and 6% at 23 IU/L. After hydrolysis with helix pomatia juice (Pall Biosepra, Cergy-Saint-Christophe, France) and extraction with diethyl ether, urinary estradiol concentration was measured by competitive immunoassay (Architect, Abbott), as also described in Peper et al. (2009). Intra-assay coefficients of variation are 9%, 3% and 4% at levels of 150, 1400 and 9000 pmol/L, respectively and inter-assay coefficient of variation is 10% for the whole range. Urinary DHEAs concentrations were measured using an in-house developed isotope dilution-liquid chromatography-tandem mass spectrometry (ID-LC-MS/MS) method, as described before, slightly adapted for measurements in urine (Büttler, Struys, Addie, Blankenstein, & Heijboer, 2012). DHEAs, LH, FSH, and estradiol levels were corrected for creatinine levels. Creatinine concentrations were measured by the Jaffé method (Modular, Roche Diagnostics, Mannheim, Germany). Inter-assay coefficients of variation are 2.2% at 5.9 mmol/L and 1.7% at 12.5 mmol/L.

Testosterone levels were measured in saliva, which provides an index of the free (i.e., unbound, or biologically available) fraction of testosterone in circulation (Rilling, Worthman, Campbell, Stallings, & Mbizva, 1996). Testosterone levels were determined using an in-house developed ID-LC-MS/MS method, as described before (Bui et al., 2013).

**MR image acquisition**

MR imaging was performed on a 3.0 Tesla GE Signa HDxt scanner (General Electric, Milwaukee, Wisconsin, USA) with a SENSE 8-channel head coil. Amongst others, the imaging protocol included a 2D echo-planar imaging sequence that was used for diffusion-weighted imaging: thirty volumes with non-collinear diffusion gradients and five images without diffusion weighting were obtained (*b*-value = 1000 mm^2^/s, TR = 13000 ms, TE = 85 ms, matrix size = 128 x 128, field of view = 256 x 256 mm, 45 slices, slice thickness = 2.4 mm). In addition, a T1-weighted image was acquired: 3D FSPGR sequence, 25cm^2^ field of view, TR of 7.8 ms, TE of 3.0 ms, slice thickness 1 mm, and 176 slices.
**MR image processing** Diffusion images were pre-processed using DTIfit, which is part of the FMRIB’s Diffusion Toolbox as implemented in the FMRIB Software Library (FSL) version 4.1.9 (Smith et al., 2004). Images were corrected for eddy current distortions, realigned to one of the non-weighted images using affine registration (Jenkinson, Bannister, Brady, & Smith, 2002), and non-brain tissue removal using BET (Smith, 2002). By fitting a tensor model to the diffusion data, the eigenvalues (λ_1_, λ_2_, λ_3_,) of the tensor for each voxel were identified, and individual FA maps were calculated.
 All participants’ FA maps were normalized via non-linear registration to the FMRIB58_FA template, and then transformed to MNI152 space using FNIRT (part of FSL). In line with the previous study in cis- and transgender adult participants (Burke, Manzouri, & Savic, 2017), we determined average FA values in 13 specific white matter tracts of interest (cortico-spinal tract, superior longitudinal fasciculus, inferior longitudinal fasciculus, inferior fronto-occipital fasciculus, each left and right side, and the forceps minor, forceps major, the splenium, corpus and genu of the corpus callosum). Anatomical locations and tracts were labelled (max. probability threshold of 25, and 1 mm isotropic voxel) using the John Hopkins University (JHU) White-Matter Tractography Atlas and JHU ICBM-DTI-81 White-Matter Labels (Hua et al., 2008; Mori, Wakana, van Zijl, & Nagae-Poetscher, 2005; Wakana et al., 2007).

**Additional Table**

Table 2. *Average fractional anisotropy (FA) values and standard deviations for all ROIs per group.*

| ***Children*** | *Cisgender boys* | *Transgender girls* | *Transgender boys* | *Cisgender girls* |
| --- | --- | --- | --- | --- |
| **L CST** | .539 (.016) | .546 (.014) | .543 (.014) | .535 (.022) |
| **R CST** | .563 (.015) | .562 (.013) | .560 (.019) | .559 (.021) |
| **Forceps major** | .501 (.023) | .508 (.021) | .506 (.015) | .496 (.019) |
| **Forceps minor** | .463 (.017) | .466 (.013) | .462 (.017) | .461 (.016) |
| **L IFOF** | .410 (.016) | .417 (.016) | .413 (.014) | .410 (.015) |
| **R IFOF** | .416 (.017) | .424 (.017) | .424 (.014) | .416 (.017) |
| **L ILF** | .311 (.015) | .313 (.015) | .308 (.011) | .310 (.017) |
| **R ILF** | .347 (.022) | .347 (.017) | .343 (.016) | .347 (.017) |
| **L SLF** | .337 (.015) | .342 (.012) | .334 (.013) | .337 (.011) |
| **R SLF** | .366 (.017) | .371 (.016) | .359 (.017) | .363 (.014) |
| **Genu of corpus callosum** | .568 (.019) | .574 (.017) | .565 (.018) | .567 (.022) |
| **Body of corpus callosum** | .489 (.026) | .494 (.021) | .481 (.018) | .486 (.021) |
| **Splenium of corpus callosum** | .550 (.018) | .552 (.015) | .545 (.013) | .545 (.016) |
|  |  |  |  |  |
| ***Adolescents*** | *Cisgender boys* | *Transgender girls* | *Transgender boys* | *Cisgender girls* |
|  |  |  |  |  |
| **L CST** | .576 (.015) | .573 (.021) | .566 (.016) | .563 (.018) |
| **R CST** | .588 (.016) | .583 (.025) | .576 (.015) | .579 (.015) |
| **Forceps major** | .547 (.017) | .539 (.026) | .543 (.020) | .548 (.023) |
| **Forceps minor** | .498 (.013) | .495 (.012) | .494 (.016) | .496 (.015) |
| **L IFOF** | .468 (.016) | .455 (.014) | .460 (.017) | .464 (.017) |
| **R IFOF** | .455 (.016) | .451 (.015) | .452 (.020) | .456 (.019) |
| **L ILF** | .361 (.013) | .361 (.020) | .364 (.018) | .365 (.015) |
| **R ILF** | .382 (.012) | .378 (.023) | .382 (.024) | .386 (.019) |
| **L SLF** | .375 (.013) | .376 (.015) | .371 (.014) | .373 (.013) |
| **R SLF** | .398 (.015) | .397 (.017) | .392 (.016) | .397 (.013) |
| **Genu of corpus callosum** | .579 (.018) | .582 (.015) | .575 (.021) | .583 (.022) |
| **Body of corpus callosum** | .520 (.022) | .514 (.022) | .512 (.021) | .527 (.028) |
| **Splenium of corpus callosum** | .596 (.015) | .591 (.021) | .594 (.018) | .597 (.020) |

Data are presented as mean (SD), range. CST = cortico-spinal tract, IFOF = inferior fronto-occipital fasciculus, ILF = inferior longitudinal fasciculus, SLF = superior longitudinal fasciculus, L = left, R = right.

**References**

Bui, H. N., Sluss, P. M., Blincko, S., Knol, D. L., Blankenstein, M. A., & Heijboer, A. C. (2013). Dynamics of serum testosterone during the menstrual cycle evaluated by daily measurements with an ID-LC-MS/MS method and a 2nd generation automated immunoassay. *Steroids, 78*, 96-101. doi:10.1016/j.steroids.2012.10.010

Burke, S. M., Manzouri, A. H., & Savic, I. (2017). Structural connections in the brain in relation to gender identity and sexual orientation. *Sci Rep, 7*, 17954. doi:10.1038/s41598-017-17352-8

Büttler, R. M., Struys, E. A., Addie, R., Blankenstein, M. A., & Heijboer, A. C. (2012). Measurement of dehydroepiandrosterone sulfate (DHEAS) in serum and cerebrospinal fluid by isotope-dilution liquid chromatography tandem mass spectrometry. *Clinica Chimica Acta, 414*, 246-247. doi:10.1016/j.cca.2012.09.024

Duke, P. M., Litt, I. F., & Gross, R. T. (1980). Adolescents' self-assessment of sexual maturation. *Pediatrics, 66*, 918-920.

Hua, K., Zhang, J., Wakana, S., Jiang, H., Li, X., Reich, D. S., . . . Mori, S. (2008). Tract probability maps in stereotaxic spaces: analyses of white matter anatomy and tract-specific quantification. *Neuroimage, 39*, 336-347. doi:10.1016/j.neuroimage.2007.07.053

Jenkinson, M., Bannister, P., Brady, M., & Smith, S. (2002). Improved optimization for the robust and accurate linear registration and motion correction of brain images. *Neuroimage, 17*, 825-841.

Kuijper, E. A. M., Houwink, E. J. F., Van Weissenbruch, M. M., Heij, H. A., Blankenstein, M. A., Huijser, J., . . . Lambalk, C. B. (2006). Urinary gonadotropin measurements in neonates: A valuable non-invasive method. *Annals of Clinical Biochemistry, 43*, 320-322. doi:10.1258/000456306777695582

Marshall, W. A., & Tanner, J. M. (1969). Variations in pattern of pubertal changes in girls. *Archives of disease in childhood, 44*, 291-303. doi:10.1136/adc.44.235.291

Marshall, W. A., & Tanner, J. M. (1970). Variations in the Pattern of Pubertal Changes in Boys. *Archives of Disease in Children, 45*, 13-23. doi:10.1136/adc.45.239.13

Mori, S., Wakana, S., van Zijl, P. C. M., & Nagae-Poetscher, L. M. (2005). MRI Atlas of Human White Matter.

Morris, N. M., & Udry, J. R. (1980). Validation of a self-administered instrument to assess stage of adolescent development. *Journal of Youth and Adolescence, 9*, 271-280. doi:10.1007/BF02088471

Rilling, J. K., Worthman, C. M., Campbell, B. C., Stallings, J. F., & Mbizva, M. (1996). Ratios of plasma and salivary testosterone throughout puberty: production versus bioavailability. *Steroids, 61*, 374-378.

Smith, S. M. (2002). Fast robust automated brain extraction. *Human Brain Mapping, 17*, 143-155. doi:10.1002/hbm.10062

Smith, S. M., Jenkinson, M., Woolrich, M. W., Beckmann, C. F., Behrens, T. E., Johansen-Berg, H., . . . Matthews, P. M. (2004). Advances in functional and structural MR image analysis and implementation as FSL. *Neuroimage, 23*, 208-219. doi:10.1016/j.neuroimage.2004.07.051

Soleman, R. S., Schagen, S. E. E., Veltman, D. J., Kreukels, B. P. C., Cohen-Kettenis, P. T., Lambalk, C. B., . . . Delemarre-van de Waal, H. A. (2013). Sex Differences in Verbal Fluency during Adolescence: A Functional Magnetic Resonance Imaging Study in Gender Dysphoric and Control Boys and Girls. *The Journal of Sexual Medicine, 10*, 1969-1977. doi:10.1111/jsm.12083

Wakana, S., Caprihan, A., Panzenboeck, M. M., Fallon, J. H., Perry, M., Gollub, R. L., . . . Mori, S. (2007). Reproducibility of quantitative tractography methods applied to cerebral white matter. *Neuroimage, 36*, 630-644. doi:10.1016/j.neuroimage.2007.02.049
